# Interaction among Cells of Bone, Immune System, and Solid Tumors Leads to Bone Metastases

**DOI:** 10.1155/2013/315024

**Published:** 2013-04-24

**Authors:** Ilaria Roato

**Affiliations:** CeRMS, A.O. Città della Salute e della Scienza, Via Santena 5, 10126 Torino, Italy

## Abstract

Bone metastases are a dismal consequence for different types of solid tumors, such as breast, prostate, lung, and kidney cancer. The mechanisms regulating the interactions among bone, immune system, and tumor cells have been deeply investigated, and many studies are ongoing to define the specific role of the different cells in the bone metastatic process. The affinity of some tumors to growth in bone results from the special microenvironment provided by bone. Moreover, immune system and bone have a bidirectional relationship: bone cells express surface molecules ruling the expansion of hemopoietic stem cells from which all cells of the mammalian immune system derive, and various immunoregulatory cytokines influence the fate of bone cells. The last findings allow to extend the concept of vicious cycle and add T cells as mediators of the tumor growth in bone.

## 1. Introduction 

Bone metastases are a common cause of morbidity in patients affected by different types of cancer and are classified in osteolytic (bone resorbing), osteoblastic (bone forming), and mixed, containing both elements. The presence of the mixed lesions suggests that the processes of bone resorption and formation may occur together and are not mutually exclusive activities. Bone metastases may occur many years after the primary tumor and have become a chronic condition for many patients with advanced cancers markedly affecting their quality of life and the demands on health care resources [1, 2]. Certain tumor types such as breast and prostate cancer show a high incidence for metastasis to bone, and a significant proportion of patients with advanced cancer of the lung, kidney, and thyroid have skeletal involvement [1]. Osteoclasts (OCs) are the main responsible for the bone destruction in osteolytic lesions, even though their activation varies depending on the tumors. OCs are multinucleated cell of hematopoietic origin residing in bone and their main activity is represented by the resorption of the mineralised bone matrix [3]. OCs attach themselves to bone, creating a microenvironment between itself and the underlying bone matrix, a specialized structure called sealing zone. This compartment is acidified by an electrogenic proton pump (H+-ATPase) and a Cl-channel in order to solubilize the mineral component of bone, exposing its organic matrix, consisting largely of type I collagen, which is subsequently degraded by cathepsin K. To facilitate the resorption process, OCs polarize their structure and form the ruffled border, which allows the active transport of H+ ions through the vacuolar proton pump [4, 5]. 

Osteoblastic metastases are prevalent in advanced prostate cancer patients and induced by cancer cell interactions with osteoblasts (OBs) and their progenitors, by production of transforming growth factor-*β* (TGF-*β*), bone morphogenetic protein, insulin-like growth factor (IGF), fibroblast growth factor (FGF), and WNTs [6]. OBs respond to morphogenetic factors by activating SMAD signalling, to growth factors by MAPK and PKC signalling, and to WNT by *β*-catenin-regulated pathways. These pathways converge on and interact with the RUNX2 transcriptional network, which drives OBs differentiation and proliferation. 

Here, I review the current knowledge on the interactions between immune system and solid tumors in promoting bone metastasis formation.

## 2. Bone and Immune System Crosstalk

Bone, hematopoietic, and immune systems are in deep physical contact and share several common pathways. OC precursors T, and B, and NK cells arise from the same stem cell, thus some of the receptors and ligands that mediate the immune process also rule the maturation of OC precursors and the ability of the mature cell to degrade bone. Immune system and bone have a bidirectional relationship: bone cells express surface molecules ruling the expansion of hemopoietic stem cells from which all cells of the mammalian immune system derive, and various immunoregulatory cytokines influence the fate of bone cells [7, 8]. OC precursors circulate among the peripheral blood mononuclear cells (PBMCs), which act as a reservoir for replenishing the pre-OC pool in the bone marrow and also as a potentially abundant source of pre-OCs that can be recruited into bone or joint tissue in response to reparative or pathological signals. 

A crucial molecular link between the immune system and bone is represented by the receptor activator of nuclear factor NF-kB ligand (RANKL), its receptor RANK, and the natural decoy receptor osteoprotegerin (OPG). The membrane RANKL is expressed by OBs/stromal cells, and the soluble RANKL is secreted by activated T cells [9, 10], whereas the receptor RANK is expressed on OC precursors and also on tumor cells [11]. The RANKL to OPG ratio in serum has been suggested to be the critical factor in determining OC activation at bone level, with higher serum RANKL to OPG ratio being a marker for upregulation of osteoclastogenesis [12]. 

The RANKL production by activated T cells can directly regulate osteoclastogenesis and bone remodelling, and it explains why different pathological conditions, such as cancer, result in systemic and local bone loss. A host of immune factors, including costimulatory receptors, cytokines such as interferon-*γ* (IFN-*γ*) and tumor necrosis factor (TNF), and T and B lymphocytes play a fundamental role in the regulation of bone cell development and bone turnover, and in pathogenesis of bone disease [13]. IFN-*γ* has a controversial role in osteoclastogenesis because it has an antiosteoclastogenic effect *in vitro* [14] and *in vivo* in animal studies [15], whereas in humans it increases in oestrogen deficiency and in rheumatoid arthritis with bone loss [16, 17]. IFN-*γ* influences osteoclastogenesis both directly and indirectly: it targets maturing OC, thus blocking OC formation [18] and it stimulates T-cell activation, thus proosteoclastogenic factors increase [19]. T cells also produce interleukin-7 (IL-7), a cytokine with different effects on hematopoietic and immunologic systems. IL-7 support B, and T lymphopoiesis [20], and it is also important for the correct bone homeostasis [21, 22]. Some studies demonstrated that IL-7 promotes osteoclastogenesis by upregulating T-cell-derived cytokines, such as RANKL [23–25] and that its production is increased by oestrogen deficiency [26]. Recently investigators focused on the OC modulatory activity of T cells, showing that OCs are able to present antigenic peptides to T cells and to induce FoxP3 expression in CD8+ T cells, which rule an inappropriate activation of the immune response [27]. The cellular responses in cell-to-cell interactions between T cells and OCs are regulated through reciprocal CD137/CD137L and RANK/RANKL interactions [28]. CD137 is a costimulatory member of the TNF receptor induced by T-cell receptor activation. Its ligand CD137L is expressed on OC precursors: *in vitro* CD137L ligation suppresses osteoclastogenesis through the inhibition of OCs precursor fusion [28]. On the other hand, RANKL expressed on T cells binds to RANK on OCs, producing a reverse signal in T cells able to enhance apoptosis.

## 3. The Interplay among Bone and Tumor Cells

**Figure 1:**
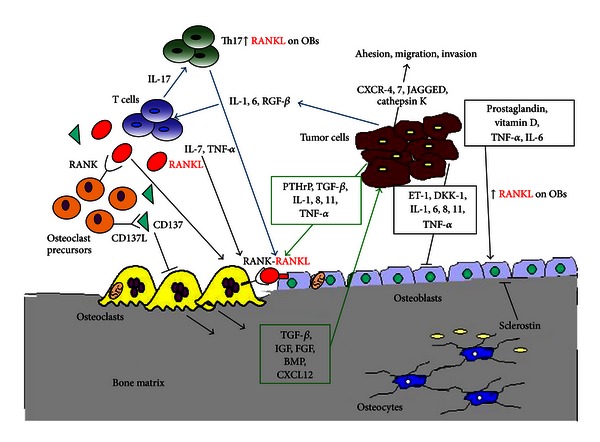
Molecular interactions among bone, tumor, and T cells.

The affinity of some tumors to growth in bone results from the special microenvironment provided by bone. These local interactions between tumor cells and bone form a vicious cycle, which underlies the development of skeletal metastases (Figure 1) [29]. Bone marrow is a favourable soil for some tumor cells, which have a biological proclivity for this tissue. For instance, bone marrow produces factors, such as CXCL12, with a chemotactic role on cancer cells, which, on the other hand, express the chemokine receptors, CXCR4 and CXCR7 [30, 31]. Moreover, activated OCs resorb bone and release growth factors enmeshed in the bone matrix, such as bone morphogenetic proteins, TGF-*β*, insulin-like growth factor, fibroblast growth factor, and others that stimulate the growth of metastatic tumor cells [32]. Cancer cells, in turn, secrete prostaglandins, parathyroid hormone, parathyroid hormone-related peptide, activated vitamin D, interleukin-6 (IL-6), and TNF that may lead to an increase in RANKL expression on OBs and bone marrow stromal cells [3], which stimulates the OC number, survival, and activity, promoting osteolytic metastases. Notch-Jagged interactions in the bone marrow suggest direct activation of osteolysis by cancer cells through this unique interaction. In particular, Jagged1, which is a downstream mediator of the prometastatic TGF-*β*, promotes tumor growth through stimulation of IL-6 production from OBs, and directly it activates OC differentiation [33]. Moreover, Jagged1 is overexpressed by bone metastatic tumor cells [34], whereas its receptor Notch is frequently expressed by progenitors and mature cells in the bone marrow [35]. In breast cancer, Notch-Jagged interactions activate biological responses in OCs and OBs, which promote both tumor invasion of bone and tumor cell growth in the bone [33].

Interestingly, prostate and breast cancer cells show osteomimicry, that is, the ability to acquire a bone cell phenotype [36–38]. Breast and prostate tumor cells respond to growth factor stimulation via activation of various osteoblastic transcription factors. This would suggest that bone lesions may also occur through differentiation of the cancer cells towards an osteoblastic bone-forming phenotype, which is a phenomenon that has been observed in the bone metastatic prostate and breast cancer celllines [39, 40]. 

## 4. T Cells as Regulator of Tumor Growth in Bone

Recently, the concept of vicious cycle has been enlarged to include T cells as additional regulators of bone tumor growth, regardless of the OC status [41]. Blockade of OC activity efficiently decreases tumor burden as well as associated bone lesions in immune-compromised animals bearing human osteolytic cancers. Despite the antiresorptive therapies are efficient in blocking the OCs, part of the treated patients develop further skeletal metastases within two years from the beginning of the treatment, suggesting that additional cells modulate bone tumor growth. These cells are T lymphocytes: tumor cell-derived IL-6, interleukin-1 (IL-1), and TGF-*β* can drive T-cell differentiation towards a Th17 secretory helper-cell phenotype capable of inducing RANKL production by OBs and OC activation through interleukin-17 (IL-17) production (Figure 1) [42]. In breast cancer patients, memory T cells have been found in the bone marrow, suggesting their role in cancer immune surveillance [43]. Moreover, the RANKL-RANK interaction between helper T cells and breast cancer cells promotes invasion, dissemination, and metastasis formation from orthotopic syngeneic mouse mammary tumor virus-Erbb2 tumors in immunocompetent mice [44]. 

Noteworthy, some antibone metastatic therapies also showed immunomodulatory effects: the blockade of TGF-*β* at metastatic sites may locally activate an antitumor T cell response because normally TGF-*β*, released in bone marrow by OC activity, inhibits T-cell proliferation [45]. Zoledronic acid, used as antiresorptive agent, can activate cytotoxic *γ*/*δ*-T cells and inhibit populations of myeloid-derived cells with T-cell-suppressor capabilities [46]. Zhang and colleagues provide compelling evidence that a condition of immune deficiency can interfere with the antitumor effects of OC blockade [41]. Modulation of antitumor T-cell response alters tumor growth in bone, indeed Lyn(−/−) mice, which have more numerous OCs and a hyperactive myeloid population with an increased T-cell responses, had reduced tumor growth in bone despite enhanced osteolysis. Lyn is a member of the Src family tyrosine kinases and it is involved in the downregulation of different intracellular pathways, including the PLC*γ*2 activation, which regulates the OC formation and function, thus Lyn inhibits OC differentiation [47]. PLC*γ*2(−/−) mice, which have dysfunctional OCs and impaired dendritic cell-mediated T-cell activation, had increased bone tumor burden despite protection from bone loss. Importantly, injection of antigen-specific wild-type cytotoxic CD8(+) T cells in both these mice models reduces the growth of tumor cells in the bone, regardless of OC functionality. According to these data, T cells seem to be critical regulators of tumor growth in bone, in particular their activation diminishes bone metastases, whereas their depletion enhances them, even in the presence of zoledronic acid. 

Further proves of the direct dialogue between T cells and OCs derive by studies conducted on the PBMCs of patients affected by solid tumors with bone metastases, which show an increase of circulating OC precursors compared with both healthy controls and cancer patients without bone metastases [48]. These OC precursors differentiate into mature, multinucleated, and bone resorbing OCs *in vitro*, without adding proosteoclastogenic factors. This osteoclastogenesis is dependent on T cells that release TNF-*α* and RANKL, which act synergistically in promoting the OC differentiation. On the other hand, T-cell depletion does not allow the differentiation of PBMCs into OCs without adding M-CSF and RANKL [48]. 

As previously reported, IL-7 is an important regulator of the interaction between bone and immune system. Many studies report a role of IL-7 in bone homeostasis in particular to bone loss in oestrogen deficiency conditions [22, 49, 50]. Other works support an active role of IL-7 in promoting bone lesions from solid tumors and multiple myeloma [24, 25, 51, 52]. In culture of PBMCs derived from patients with bone metastases, IL-7 is mainly released by B cells, and it directly sensitises T cells to produce proosteoclastogenic factors, such as TNF-*α* and RANKL, which enhance spontaneous osteoclastogenesis [25]. Moreover, in bone metastatic patients IL-7 sera levels were found significantly higher than in nonbone metastatic patients and in healthy controls [25, 52]. This serum IL-7 increase seems to directly depend on tumor production: a strong IL-7 expression was detected in tumor masses originated in a human-in-mice model of bone metastases and in human bone metastatic biopsies [53]. 

Prostate cancer is typically characterised by the presence of osteoblastic lesions with underlying osteolytic area [54]. The osteolytic activity is explained by an increase in serum RANKL/OPG ratio in prostate cancer patients, thus an enhanced OC formation plays an active role in bone forming lesions. The RANKL increment depends at least in part on the increased IL-7 production from T and B cells, but unlike other solid tumors, IL-7 expression is not significantly different in prostate cancer patients and in normal controls [51]. In the early phase of prostate cancer bone metastases, the increased OC activity also depends on Wnt agonist Dickkopf-1 (DKK-1) that inhibits OBs, favouring lytic lesions [55]. In a subsequent phase, there is an increase in endothelin-1 (ET-1), which stimulates OBs and inhibits OCs, by decreasing the transcription of DKK-1 [56]. ET-1 is also expressed by breast cancer cells, explaining the presence of mixed bone lesions in advanced disease. 

## 5. Molecular Targets for the Treatment of Bone Metastases

In the last years, thanks to the identification of the above reported factors produced by bone, tumor, and immune system cells, many novel agents able to prevent or block bone metastases have been developed. The identification of the RANKL/RANK/OPG signalling pathway provided the basis for the development of new therapeutic molecules, such as denosumab, the first fully human antibody anti-RANKL. The first attempt to interfere with RANKL signalling to block and/or prevent bone metastases was the use of OPG or OPG-Fc [57, 58]. Unlike OPG-Fc, denosumab specifically binds to RANKL, without interfering with other TNF ligands. In clinical trials, it resulted able to prevent the homing of RANK expressing tumor cells to bone and to inhibit the formation of bone lesions [59], thus it has been approved for the treatment of bone metastases in prostate and breast cancer patients. Even though denosumab inhibits bone resorption in patients refractory to bisphosphonate therapy [60] and ameliorates the quality of life of patients bearing bone metastases, it dose not improve the overall survival of these patients has not been demonstrated.

Src (sarcoma) is a protooncogene encoding a tyrosine kinase; it is highly expressed on OCs, where it is activated, during the bone resorption, in the process of RANK signalling and after the integrin binding. Src signalling is essential for the organization of OC cytoskeleton [61] and its lack does not allow to build an intact ruffled border causing osteopetrosis [62]. Src also inhibits RUNX2-regulated genes, thus it negatively regulates OBs [63]. Moreover, increased Src expression and activity associated with metastatic ability of many tumors [64]. Due to Src action on OCs, OBs and cancer cells, several Src inhibitors are currently being evaluated in clinical trials to test their ability in blocking skeletal lesions [65, 66].

Cathepsin K is expressed by mature OCs, being fundamental for their resorptive acitivity. It is expressed in different tumors [67, 68] and it promotes osteolytic lesions in a mouse model of prostate cancer, whereas its inhibition reduces the skeletal lesion in a breast cancer model [69]. Cathepsin K inhibitors have been investigated and tested for bone metastases treatment [70], but currently there are ongoing clinical trials only for its use in osteoporosis. 

Activin A, a member of the TGF-*β* family, has been described to stimulate osteoclastogenesis and to inhibit OBs. Increased levels of activin A have been described in the bone marrow plasma of breast and prostate cancer with bone metastases [71]. In mouse models of human breast cancer and multiple myeloma, activin A inhibition is effective to prevent bone lesions [72, 73].

DKK-1 is inhibitor of Wnt signalling pathway that regulates OB development and activity and indirectly osteoclastogenesis by shifting the RANKL-to-OPG ratio towards OPG [74]. The inhibition of DKK-1 could be an effective method to prevent osteolytic bone lesions, thus its inhibitor has been tested with encouraging results in experimental model of multiple myeloma and breast cancer [75, 76]. An anti- DKK-1 antibody (BHQ-880) is currently under investigation in three clinical trials. 

Inhibition of ET-1 signalling is a target for the inhibition of osteoblastic metastases in advanced prostate cancer, and both anti-ET-1 antibodies and blockade of ET-A receptor have been proposed [77, 78].

## 6. Conclusion

It has become increasingly evident that tumor cells interact with bone microenvironment and induce the activation of the immune system cells, which in turn release many factors able to promote bone metastases (Figure 1). This interplay creates cellular feedback loops with activation of multiple signalling, providing different potential molecular targets for the prevention and treatment of bone metastases.
